# Identification of prognostic biomarkers for papillary thyroid carcinoma by a weighted gene co‐expression network analysis

**DOI:** 10.1002/cam4.4602

**Published:** 2022-02-12

**Authors:** Kexin Meng, Xiaotian Hu, Guowan Zheng, Chenhong Qian, Ying Xin, Haiwei Guo, Ru He, Minghua Ge, Jiajie Xu

**Affiliations:** ^1^ Otolaryngology& Head and Neck Center, Cancer Center, Department of Head and Neck Surgery Zhejiang Provincial People's Hospital (Affiliated People's Hospital, Hangzhou Medical College) Hangzhou Zhejiang China; ^2^ Key Laboratory of Endocrine Gland Diseases of Zhejiang Province Hangzhou Zhejiang China; ^3^ Qingdao University Qingdao Shandong China; ^4^ Bengbu Medical College Bengbu China; ^5^ School of Basic Medical Sciences and Forensic Medicine, Hangzhou Medical College Hangzhou China

**Keywords:** biomarker, overall survival, papillary thyroid carcinoma, weighted gene co‐expression network analysis

## Abstract

**Aim:**

Whole transcriptome analysis was conducted to identify differentially expressed RNAs and regulatory networks associated with papillary thyroid carcinoma (PTC).

**Methods:**

A weighted gene co‐expression network analysis based on high‐throughput sequencing data for six pairs of PTC and adjacent tissue samples was conducted to understand the biological functions and regulatory networks involving long non‐coding RNAs (lncRNAs), circular RNAs (circRNAs), microRNAs (miRNAs), and messenger RNAs (mRNAs).

**Results:**

We detected 131, 338, 31, and 556 differentially expressed circRNAs, lncRNAs, miRNAs, and mRNAs, respectively. We identified modules that were significantly positively and negatively related to cancer and lymph node metastasis. Gray and turquoise modules were positively correlated with cancer phenotypes (*p* < 0.05), whereas yellow, brown, and blue modules were negatively correlated with cancer (*p* < 0.05). Gray module was positively correlated with lateral lymph node metastasis (*p* = 0.02). Kaplan–Meier analyses revealed that the levels of transmembrane protein 63C (*TMEM63C*), lysyl oxidase‐like 1 (*LOXL1*), collagen type V alpha 1 chain (*COL5A1*), ADAM metalloproteinase with thrombospondin type I motif 2 (*ADAMTS2*), and LysM‐domain containing 3 (*LYSMD3*) were significantly associated with overall survival (*p* < 0.05). Significant increase in the expression of *COL5A1* and *LOXL1* in tumor tissues was validated by quantitative real‐time polymerase chain reaction (*p* < 0.05). *COL5A1* and *LOXL1* promoted PTC cell growth and invasion in vitro.

**Conclusions:**

We identified *COL5A1* and *LOXL1* as potential prognostic biomarkers, providing new insights into the occurrence and progression of PTC.

## Graphical abstract

Whole transcriptome analysis was conducted to identify differentially expressed RNAs and regulatory networks associated with papillary thyroid carcinoma (PTC). A weighted gene co‐expression network analysis based on high‐throughput sequencing data for six pairs of PTC and adjacent tissue samples was conducted to understand the biological functions and regulatory networks involving long non‐coding RNAs (lncRNAs), circular RNAs (circRNAs), microRNAs (miRNAs), and messenger RNAs (mRNAs).

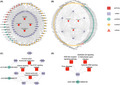

## 1 INTRODUCTION

Papillary thyroid carcinoma (PTC) is the most common endocrine malignancy. GLOBOCAN recently reported that the global incidence rate of PTC is 3%, with an incidence rate of 4.9% for women, ranking 5th in tumor incidence worldwide.^1^ Although the overall prognosis of PTC is good, it has been reported that 5%–21% of the patients experience tumor recurrence, lymph node metastasis, or post‐surgery distant metastasis, thus contributing to a significantly reduced survival rate.^2^ Cervical lymph node metastasis^3^, ^4^ and extrathyroidal extension invasion^4^ are significantly associated with PTC recurrence. Some PTC cases show rapid progression, presenting tracheal, and nerve invasion at the initial diagnosis^5^; therefore, extensive surgery is required in these cases to minimize recurrence.^6^, ^7^ This leads to an increase in postoperative complications, such as recurrent laryngeal nerve palsy, permanent hypoparathyroidism, and increased cervical paresthesia, which worsen the quality of life of the patients.^8^ Further investigation of the genetic and regulatory mechanisms underlying PTC metastasis are, thus, of great importance with respect to the evaluation of high‐risk patients and for the development of effective interventions, treatments, and prognostic markers.

Continued advances in high‐throughput sequencing (RNA‐seq) technology and bioinformatic approaches have led to the discovery of various non‐coding RNAs (ncRNAs), such as circular RNAs (circRNAs) and long non‐coding RNAs (lncRNAs). ncRNAs do not directly encode proteins. Instead, they participate in gene regulation via various mechanisms. The interplay among circRNAs, lncRNAs, and microRNAs (miRNAs) can directly or indirectly regulate tumor development. Whole transcriptome analyses and the construction of co‐expression networks can reveal potential regulatory mechanisms involved in PTC development, invasion, and metastasis.

Weighted gene co‐expression network analysis (WGCNA) based on RNA‐seq data is used to explore the relationships between gene networks and diseases as well as correlations between gene modules and clinical characteristics.^9^, ^10^ In WGCNA, genes with similar biological functions are classified into the same module by a module‐clinical trait relationship analysis.^11^ This approach matches genes with clinical characteristics, allowing researchers to identify key genes for follow‐up studies, thus significantly improving research efficiency.^12^

In this study, WGCNA was used to analyze high‐throughput sequencing data for six pairs of PTC and adjacent non‐neoplastic tissue samples to understand the biological functions contributing to PTC development and progression and to identify potential co‐expression networks of lncRNAs, circRNAs, miRNAs, and messenger RNA (mRNAs). Furthermore, candidate survival‐related genes and their functions were evaluated. These results improve our understanding of ncRNA interactions associated with the occurrence and development of PTC, thereby contributing to the development of prognostic markers and treatment strategies.

## 2 MATERIALS AND METHODS

### 2.1 Sample collection

In total, six pairs of PTC tissues and adjacent non‐neoplastic tissues for RNA‐seq and 18 pairs of samples for expression validation, were collected from patients with PTC who underwent thyroidectomy between December 2019 to June 2021 at the Department of Head and Neck Surgery, Zhejiang Provincial People's Hospital (Hangzhou, China). All patients provided written informed consent before surgery. After immediate snap freezing in liquid nitrogen post‐resection, all specimens were stored at ˗80 °C until further use. This study was approved by the Ethics Committee of Zhejiang Provincial People's Hospital and conducted in accordance with the Declaration of Helsinki (Approval No. 2019KY298).

### 2.2 RNA isolation

Total RNA was extracted using TRIzol reagent (Invitrogen) in accordance with the manufacturer's protocol. DNase I (New England Biolabs) treatment was employed to degrade any remaining DNA. The quality and quantity of isolated RNAs were assessed using the NanoDrop 2000 (Thermo Fisher Scientific). RNA with A_260_/A_280_ ≥ 1.6 was used for subsequent experiments.

### 2.3 Whole transcriptome library synthesis and sequencing

The Ribo‐Minus Kit (Life Technology) was used to deplete ribosomal RNA (rRNA) from purified RNA following the manufacturer's protocols. The rRNA‐depleted RNA samples were then fragmented, and complementary DNA (cDNA) was synthesized according to the manufacturer's instructions (Illumina). After purifying the PCR amplification products of cDNA, the libraries were subjected to quality control and sequencing using the HiSeq2500 system (Illumina) to obtain 2 × 150 bp paired‐end reads. Workflow of transcriptome sequencing data analysis was shown in supplemental document.

### 2.4 Differential expression analysis

The R package “edgeR”^13^ (version 3.14, http://bioconductor.org/packages/release/bioc/html/edgeR.html) was used to identify differentially expressed genes (DEGs) by pairwise comparisons in R (version 4.10). A criterion of *p* < 0.05 was used to identify significant DEGs.

### 2.5 Construction of weighted gene co‐expression networks and identification of modules associated with phenotypes

We normalized the expression of mRNA, miRNA, circRNA, and lncRNA in six neoplastic and adjacent tissues and constructed weighted gene co‐expression networks of DEGs for the 12 PTC samples using the R package WGCNA (version 1.70, https://cran.r‐project.org/web/packages/WGCNA/index.html). When the soft‐thresholding power was equal to 16, the correlation coefficient reached 0.9, and a scale‐free co‐expression network was obtained.

Genes with similar expression patterns were clustered into the same modules by hierarchical clustering dendrograms. The minimum size of each module was set at 30 genes. The optimal cutoff height was set to 0.25, and six modules were generated. Since these modules were composed of gene sets and not individual variables, correlations with clinical phenotypes could not be directly evaluated. Module eigengene (ME) was defined as the first principal component within the module, as it is able to quantify the expression of all co‐expressed genes in the module. Relationships between MEs and clinical phenotypes were analyzed by module–trait associations, and the results were visualized using a heatmap.

### 2.6 Functional enrichment analysis of genes in every module

Module functions were analyzed using the R package “clusterProfiler” (version 3.14, https://bioconductor.org/packages/release/bioc/html/clusterProfiler.html). The Kyoto Encyclopedia of Genes and Genomes (KEGG) database and Gene Ontology (GO) biological processes were used for functional enrichment analyses. *p* < 0.05 was regarded as the cutoff for significance.

### 2.7 Co‐expression network construction

A co‐expression network of genes in key co‐expression modules was imported into Cytoscape (version 3.8.2, https://cytoscape.org/) to construct and visualize a lncRNA–circRNA–miRNA–mRNA network of the module. Hub genes were determined according to the node degree, and the top 20 genes in each module were selected.

### 2.8 Survival analysis

Kaplan–Meier analyses and Cox regression analyses were used to assess the associations between hub genes and survival. Overall survival (OS) and progression‐free survival (PFS) data were obtained from The Cancer Genome Atlas (TCGA) database. A Kaplan–Meier analysis of the hub genes was performed and significance was evaluated using the log‐rank test. To define high‐ and low‐risk groups of the hub genes, the optimal cutoff value was selected based on the maximal survival difference at the lowest log‐rank *p*‐value using the surv cutpoint function of the R package “survminer”^14^ (version 0.49, https://cran.r‐project.org/web/packages/survminer/index.html). Cox regression analyses were used to analyze TCGA data from LinkedOmics (http://www.linkedomics.org/login.php).^15^

### 2.9 Quantitative reverse transcription‐polymerase chain reaction (qRT‐PCR)

Total RNA was extracted and reverse transcribed into cDNA using the PrimeScript RT Reagent Kit (Takara) under the recommended conditions. qRT‐PCR was performed using the Applied Biosystems 7500 with Hieff UNICON® Power qPCR SYBR Green Master Mix (Yeasen). β‐Actin was used as the internal reference. Each sample test was technically replicated in triplicate. The primers are listed in Table S1. The relative expression levels of the genes were calculated using the 2^−ΔΔCT^ method.^16^

### 2.10 Cell culture and transfection

The human PTC cell line TPC‐1 was kindly provided by Dr. Xin Zhu^17^ (Zhejiang Cancer Hospital). To verify the authenticity of TPC‐1 cell lines, DNA short tandem repeat (STR) analysis was performed by Biowing. TPC‐1 cells were cultured in RPMI‐1640 (Gibco) containing 10% fetal bovine serum (FBS; Gibco). TPC‐1 cells were cultured at 37°C and 5% CO_2_ for 24 h before transfection. Small interfering RNAs (siRNAs) against *LOXL1* and *COL5A1* and siRNA negative control (NC) oligonucleotides were designed by Hanbio. The sequences of siCOL5A1, siLOXL1, and siRNA NC are listed in Table S2. For transient transfection, TPC‐1 cells were transfected with siRNAs at 30% confluence using Lipofectamine 3000 (Invitrogen) according to the manufacturer's instructions.

### 2.11 Transwell invasion assay

For invasion assays, 7 × 10^4^ tumor cells in 300 μl of serum‐free RPMI 1640 medium were seeded in the upper chambers of a transwell (Coming Glass Works) coated with Matrigel (BD Biosciences) at 1:10 dilution. Then, 700 μl of 20% FBS and RPMI 1640 medium were added to the lower chambers and cells were allowed to migrate toward the lower chamber for 24 hours.

The membranes were fixed with paraformaldehyde and stained with crystal violet (Beyotime). Cells on the upper surface of the filter were removed by wiping with a small wet cotton swab, and the cells that migrated through the membrane were photographed and quantified using ImageJ (version 1.8.0, https://imagej.nih.gov/ij/). These experiments were performed in biological triplicate, and three images were processed per membrane.

### 2.12 Cell Counting Kit‐8 assay

Cell proliferation was measured using the Cell Counting Kit‐8 (CCK‐8; APExBIO). Three thousand cells were seeded in 96‐well plates with 100 μl of culture medium per well, and each treatment group had three independent replicates. After 24 h of incubation at 37 °C, 10 μl of the CCK‐8 reagent was added to each well at various time points (0, 24, 48, and 72 h). Viable cells were counted by measuring Absorbance_450_. The assays were technically replicated in triplicate.

### 2.13 Statistical analyses

SPSS v.22.0 (IBM Corp.), the R package “ggplot2” (version 3.3.5, https://ggplot2.tidyverse.org/), and GraphPad Prism 9 (GraphPad Software Inc.) were used for statistical analyses and the generation of plots. All data are presented as mean ± standard deviation (SD). Differences between groups were evaluated using the Student's *t*‐test and analysis of variance. Statistical significance was set at *p* < 0.05.

## 3 RESULTS

### 3.1 Screening of DEGs

**FIGURE 1:**
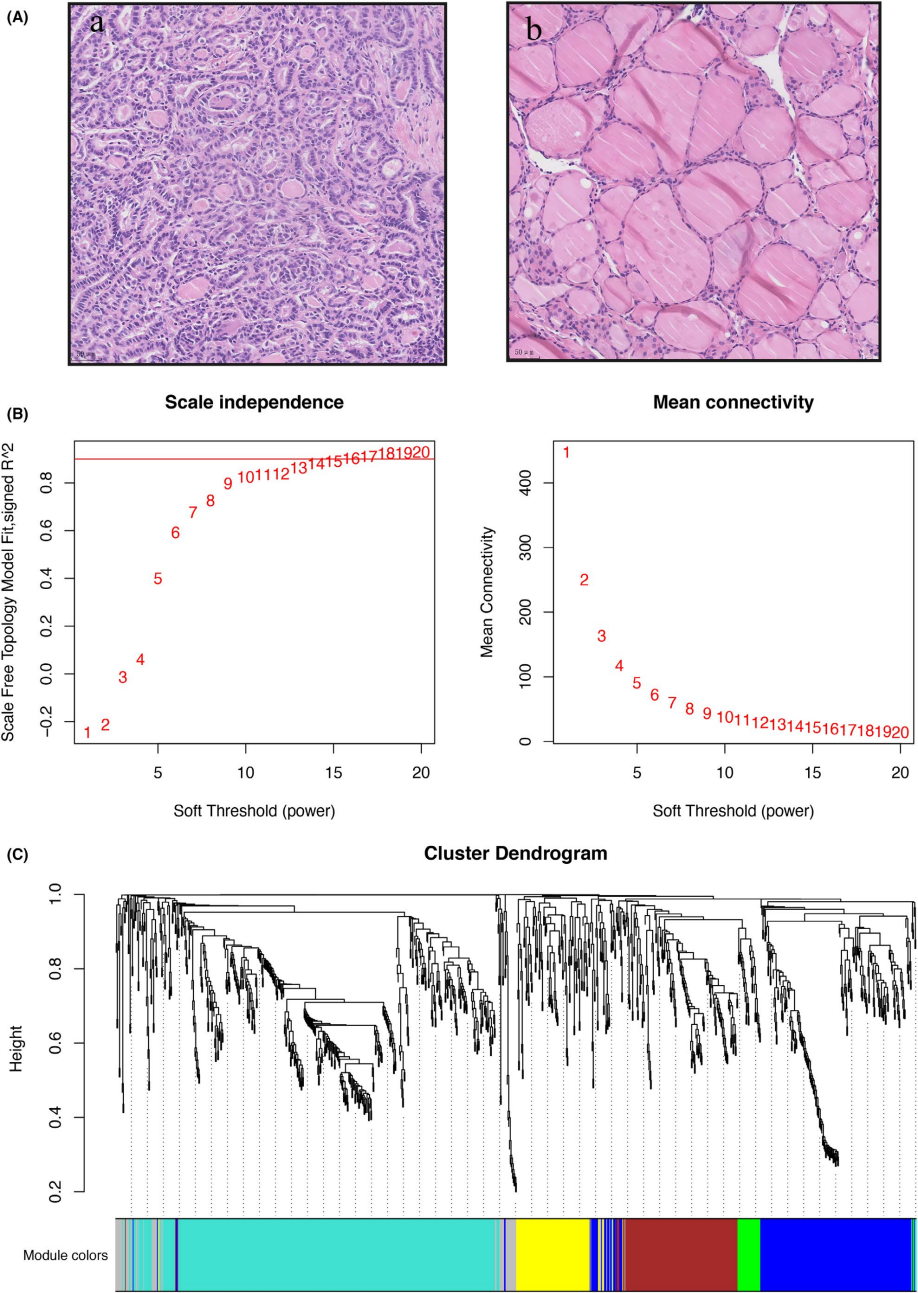
(A) representative H&E‐stained sections of papillary thyroid carcinoma tissues (a) and cancer‐adjacent tissues (b). Scale bar, 50 μm. (B) Identification of gene modules and construction of a weighted gene co‐expression network. A soft‐threshold power of 16 was used for an approximate scale‐free topology. (C) A gene dendrogram revealed six modules labeled by different colors

Details of the six pairs of PTC and cancer‐adjacent tissues are provided in Figure 1A and Table S3. The expression profiles of circRNAs, lncRNAs, miRNAs, and mRNAs were determined by RNA‐seq. DEGs were screened using the edgeR package, with a *p*‐value threshold of <0.05. We found 131 differentially expressed (DE) circRNAs, of which 25 were upregulated and 106 were downregulated in PTC, 338 DE lncRNAs, of which 47 were upregulated and 291 were downregulated in PTC, 31 DE miRNAs, of which 20 were upregulated and 11 were downregulated in PTC, and 556 DE mRNAs, of which 413 were upregulated and 143 were downregulated in PTC (Figure S1A–D). The expression levels of these genes in cancer and cancer‐adjacent tissues were plotted using a heatmap (Figure S2A–D).

### 3.2 Construction of a weighted gene co‐expression network

**FIGURE 2:**
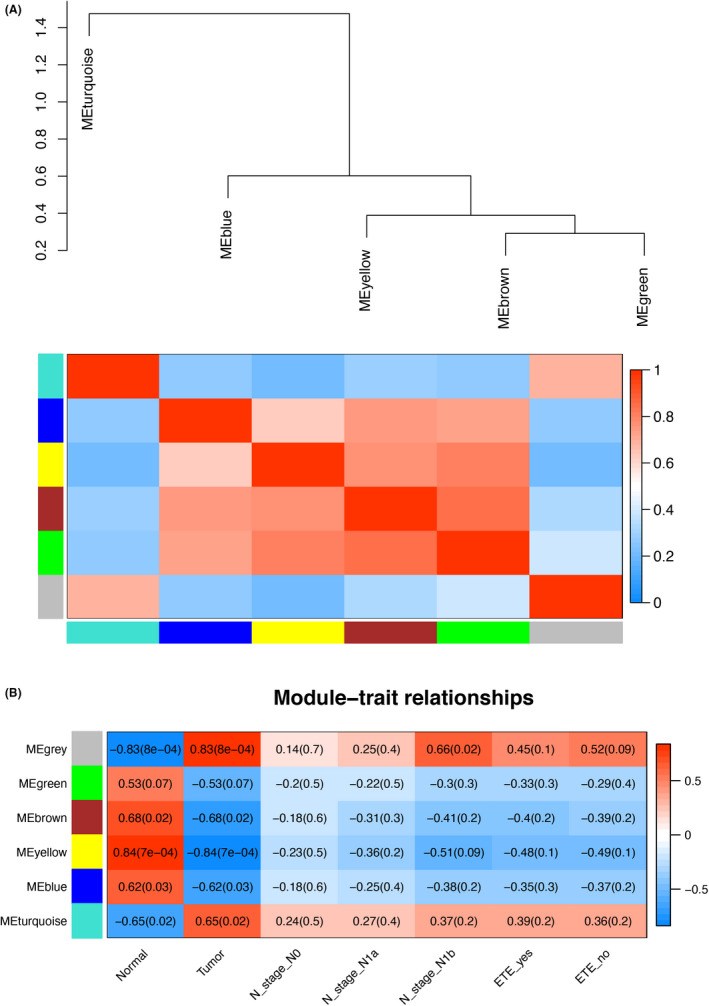
(A) Heatmap of correlations between different modules. Red represents a positive correlation and blue represents a negative correlation. (B) Relationships between module eigengenes and different phenotypes. ETE: extrathyroidal extension

The DE circRNAs, lncRNAs, miRNAs, and mRNAs were used to construct a weighted gene co‐expression network. When the soft threshold β was 16, six merged co‐expression modules were constructed by WGCNA (Figure 1B). These modules were labeled gray, green, brown, yellow, blue, and turquoise and contained different numbers of co‐expressed genes (Figure 1C). For each module, the co‐expression genes were summarized by ME. To investigate the correlations between phenotypes and MEs as well as the connectivity of MEs (Figure 2A), module–trait relationships were evaluated (Figure 2B). Gray and turquoise modules were positively correlated with the cancer phenotype, whereas yellow, brown, and blue modules were negatively correlated with cancer (*p* < 0.05, Figure 2B), and green module was not correlated with cancer (*p* = 0.07). In a correlation analysis of lymph node metastasis, we found that gray module was positively correlated with the N1b stage (*p* = 0.02, Figure 2B). No correlations were identified between the six modules and lymph node N1a stage or extrathyroid extension (ETE).

### 3.3 Gene module enrichment analysis

**FIGURE 3:**
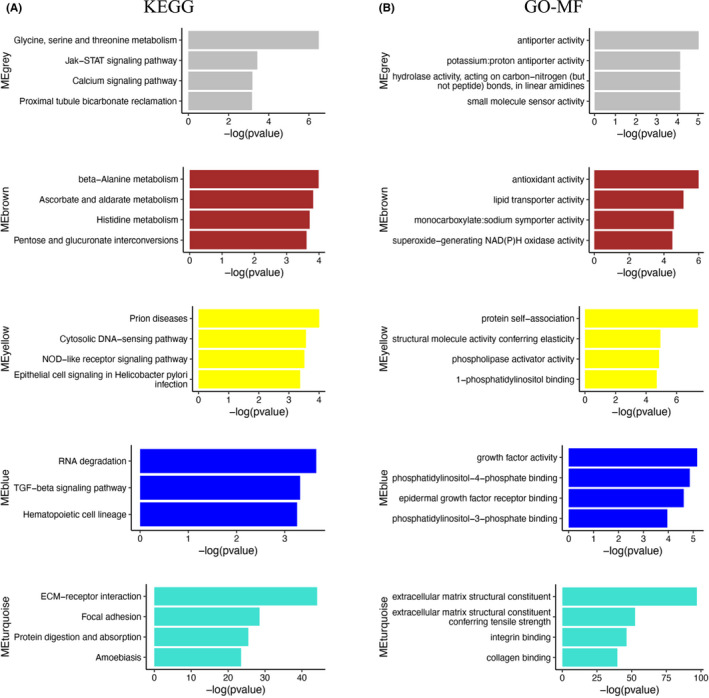
(A) Kyoto Encyclopedia of Genes and Genomes (KEGG) pathway analyses of top four pathways related to the gray, brown, yellow, blue, and turquoise modules. (B) Gene Ontology (GO) enrichment analyses of top three or four functions related to the gray, brown, yellow, blue, and turquoise modules

To investigate the main functions of the genes and candidate signaling pathways related to PTC development, we performed GO and KEGG analyses of genes in gray, turquoise, yellow, brown, and blue modules, which were closely related to cancer traits. GO analysis revealed that the main functions of the gray module were related to antiporter activity, the turquoise module was enriched for extracellular matrix (ECM) structural constituents and integrin binding, and the brown module was associated with antioxidant activity and lipid transporter activity (Figure 3B). KEGG pathway analysis showed that genes in gray module were mainly involved in glycine, serine, and threonine metabolism, and the JAK–STAT pathway, genes in turquoise module were mainly related to ECM receptor interactions, and genes in blue module were mainly involved in the transforming growth factor (TGF)‐β signaling pathway (Figure 3A).

### 3.4 Construction of molecular regulatory networks of functional genes in modules

**FIGURE 4:**
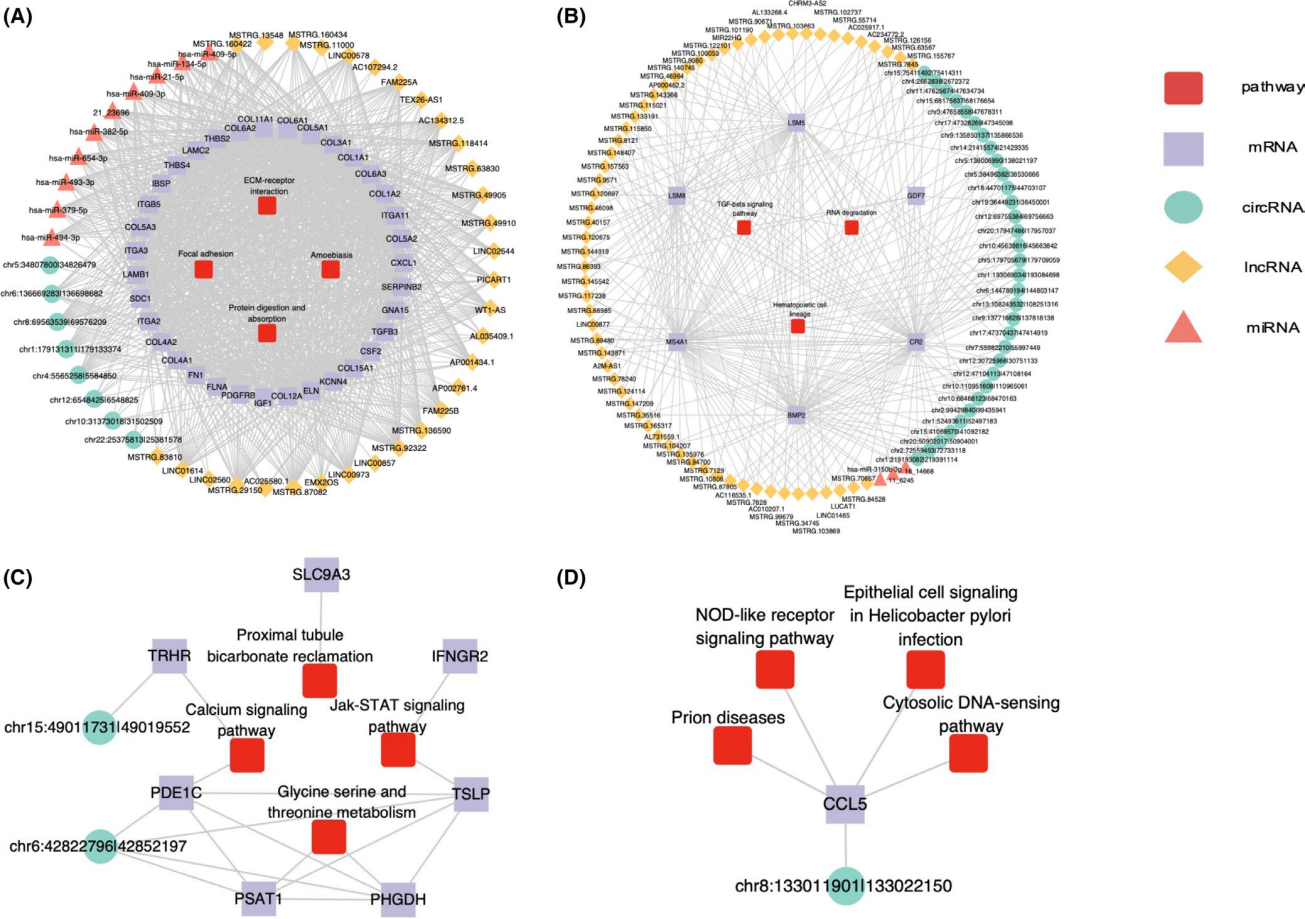
Co‐expression network of circular RNAs, long non‐coding RNAs, microRNAs, and messenger RNAs for each module associated with the top four enriched pathways. (A) turquoise module, (B) blue module, (C) gray module, and (D) yellow module

To gain insights into molecular interactions, we constructed co‐expression networks of circRNAs, lncRNAs, and miRNAs with mRNAs in each module. In four modules (i.e., turquoise, blue, gray, and yellow), we found that the genes may be regulated by at least one other molecule (e.g., lncRNA/circRNA/miRNA) (Figure 4). Cytoscape was used to visualize the network for each module, and the top 20 hub genes in each module are listed in Table S4.

### 3.5 Screening of survival‐related genes

**FIGURE 5:**
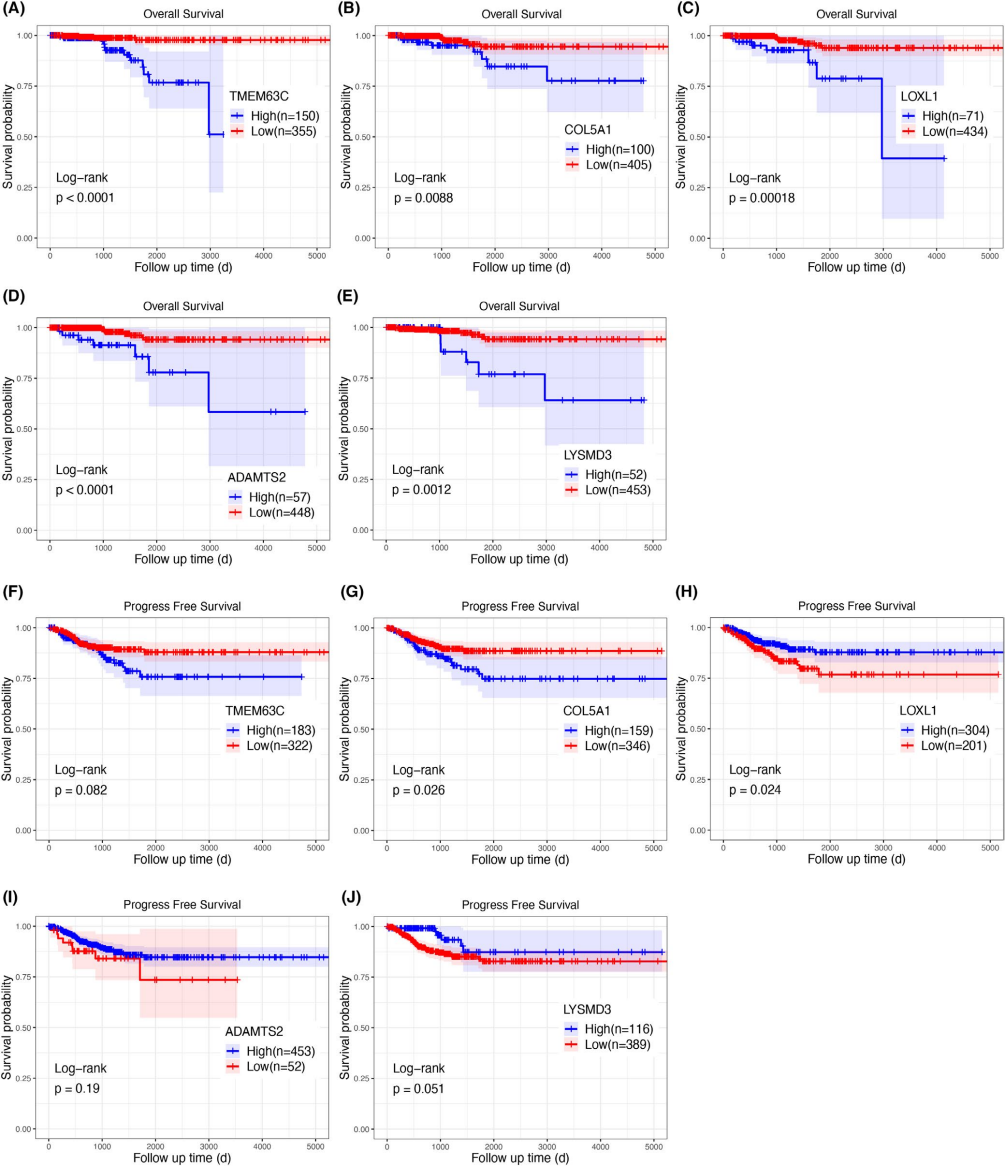
Kaplan–Meier curves of overall survival (A–E) and progression‐free survival (F–J) for expression levels of five genes (transmembrane protein 63C (*TMEM63C*), lysyl oxidase‐like 1 (*LOXL1*), collagen type V alpha 1 chain (*COL5A1*), ADAM metalloproteinase with thrombospondin type I motif 2 (*ADAMTS2*), and LysM‐domain containing 3 (*LYSMD3*))

Hub genes in gray, turquoise, yellow, brown, and blue modules were used to conduct survival analyses using TCGA survival data. In Kaplan–Meier analyses of OS, high expression levels of transmembrane protein 63C (*TMEM63C*) in gray module, lysyl oxidase‐like 1 (*LOXL1*), collagen type V alpha 1 chain (*COL5A1*), and ADAM metalloproteinase with thrombospondin type I motif 2 (*ADAMTS2*) in turquoise module, and LysM‐domain containing 3 (*LYSMD3*) in blue module were associated with a poorer prognosis than that for low expression levels (*p* < 0.05, Figure 5A–E). There were no survival‐related genes in brown and yellow modules.

In Kaplan–Meier analyses of PFS, a high expression level of *COL5A1* indicated an unfavorable outcome, whereas a high expression level of *LOXL1* was related to a favorable outcome (*p* < 0.05, Figure 5F–J). Expression levels of *TMEM63C, ADAMTS2,* and *LYSMD3* were not associated with PFS.

Cox regression analysis of OS showed that *TMEM63C*, *LOXL1*, *COL5A1, ADAMTS2,* and *LYSMD3* levels were significantly associated with poor prognosis (*p* < 0.05, Table 1). These results were in accordance with those of Kaplan–Meier analyses.

**TABLE 1:** Cox regression test of survival significant hub genes in gray, blue, and turquoise

Module	Gene name	Cox Coefficient	*p* value	FDR corrected
Gray	*TMEM63C*	0.6481	0.0004249	0.0007891
Blue	*LYSMD3*	1.157	0.044570	0.1095
Turquoise	*LOXL1*	0.4522	0.03666	0.05957
	*COL5A1*	0.2788	0.03683	0.05985
	*ADAMTS2*	0.3234	0.01905	0.03539

Abbreviations: *ADAMTS2*, ADAM metalloproteinase with thrombospondin type I motif 2; *COL5A1*, collagen type V alpha 1 chain; FDR, false discovery rates; *LOXL1*, lysyl oxidase‐like 1; *LYSMD3*, LysM‐domain containing 3; *TMEM63C*, transmembrane protein 63C.

Based on the above results, we can hypothesize that *COL5A1* and *LOXL1* in the turquoise module may be involved in the tumorigenesis of PTC via the ECM‐related pathways and are closely related to prognosis.

### 3.6 Validation of survival‐related genes in PTC tissues

**FIGURE 6:**
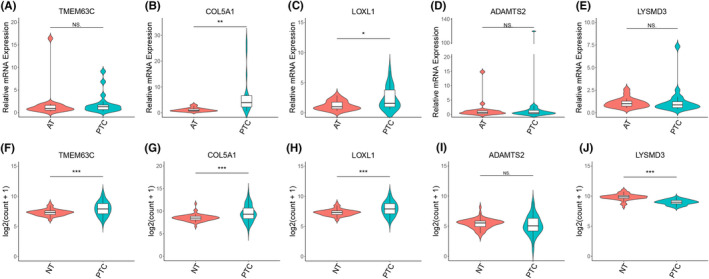
Validation of expression patterns for five genes (*TMEM63C, COL5A1*, *LOXL1*, *ADAMTS2,* and *LYSMD3*) in papillary thyroid carcinoma samples (A–E) by quantitative reverse transcription‐polymerase chain reaction (qRT‐PCR) and (F–J) by an analysis of paired samples (n = 59) from The Cancer Genome Atlas (TCGA) database. AT: adjacent tissue. PTC: papillary thyroid carcinoma. NT: normal tissue. *p < 0.05; **p < 0.01; ***p < 0.001; ns, not significant

The elevated expression levels of the survival‐related genes (i.e., *TMEM63C*, *COL5A1*, *LOXL1*, *ADAMTS2,* and *LYSMD3*) in PTC tissues were further validated by qRT‐PCR. *COL5A1* and *LOXL1* expression levels were significantly higher in 18 paired tumor tissues than in cancer‐adjacent tissues (*p* < 0.05, Figure 6A–E), consistent with the results obtained by RNA‐seq (Table 2).

**TABLE 2:** Differentially expressed genes of survival related hub gene

Module	Gene name	Fold change	*p* value
Gray	*TMEM63C*	−1.57532	0.019481
Blue	*LYSMD3*	−0.93201	0.02309
Turquoise	*LOXL1*	1.792383	0.003264
	*COL5A1*	2.685652	0.000117
	*ADAMTS2*	2.055023	0.00057

Expression data from TCGA were extracted for further analyses. Paired malignant and normal PTC tissue samples were available for 59 patients. *COL5A1, LOXL1,* and *TMEM63C* expression levels were significantly higher and *LYSMD3* expression levels were significantly lower in tumor tissues than in the paired normal tissues (Figure 6F–J). The results for *COL5A1, LOXL1,* and *LYSMD3* expression levels were in accordance with RNA‐seq results. However, there was no difference in *ADAMTS2* expression between tumor and normal tissues.

In unpaired samples, the expression levels of *COL5A1* and *LOXL1* were significantly higher in thyroid cancer than in normal tissues (505 cancer and 59 normal samples), and *LYSMD3* levels were significantly lower in thyroid cancer (Figure S3).

### 3.7 Effects of *COL5A1* and *LOXL1* on PTC cell growth

**FIGURE 7:**
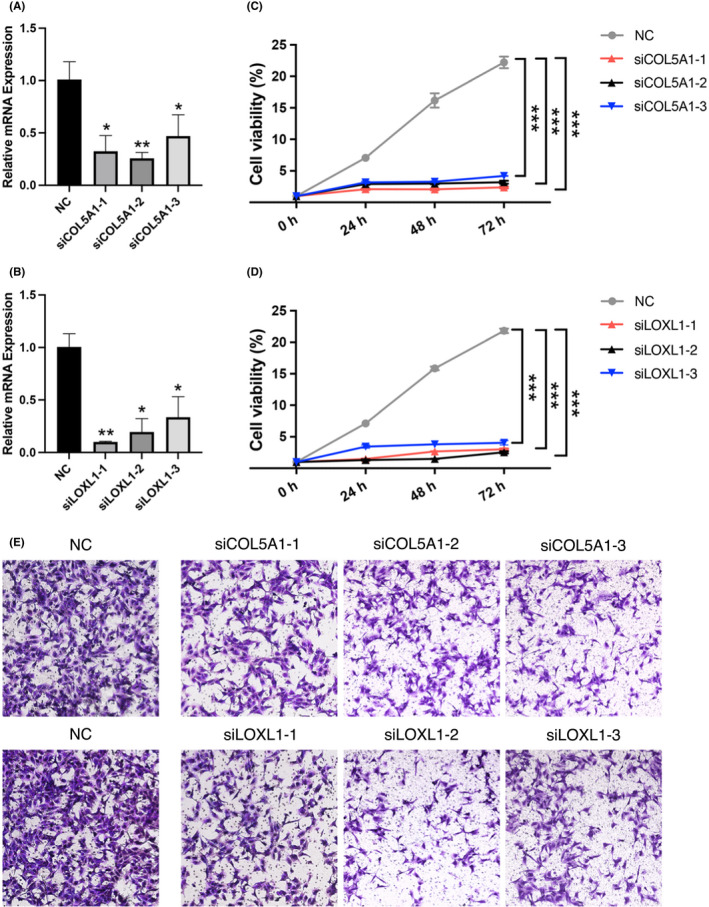
Knockdown of *COL5A1* and *LOXL1* inhibited TPC‐1 cell growth and invasion. Decreased mRNA levels of *COL5A1* (A) and *LOXL1* (B) in TPC‐1 cells after siRNA transfection by quantitative reverse transcription‐polymerase chain reaction (qRT‐PCR). Cell Counting Kit‐8 assay showed that downregulated expression of *COL5A1* (C) and *LOXL1* (D) significantly inhibited the proliferation of TPC‐1 cells. (E) Transwell invasion assay indicated that the knockdown of *COL5A1*and *LOXL1* inhibited TPC‐1 cell migration. *p < 0.05; **p < 0.01;***p < 0.001

*COL5A1* and *LOXL1* expression was upregulated in PTC, as verified by qRT‐PCR; therefore, we chose the two genes for further functional analyses. *COL5A1* and *LOXL1* expression was lower in TPC‐1 cells after transfection with siCOL5A1 and siLOXL1, as determined by qRT‐PCR (Figure 7A,B). In a CCK8 assay, the proliferation ability of TPC‐1 cells was lower in the transfection group than in the control group (Figure 7C,D).

### 3.8 Effects of *COL5A1* and *LOXL1* on PTC cell invasion

Based on the transwell invasion assay, the invasion abilities of TPC‐1 cells were remarkedly reduced in response to the downregulation of *COL5A1* (Figure 7E). A similar result was obtained after the downregulation of *LOXL1* (Figure 7E).

These results demonstrated that the downregulation of *COL5A1* and *LOXL1* significantly inhibited the proliferation and migration of PTC cells.

## 4 DISCUSSION

The WGCNA algorithm is commonly used for gene co‐expression network analyses. A soft threshold is used to avoid the potential loss of data.^18^ In recent years, WGCNA has been applied in PTC cancer and has emerged as a promising tool for the identification of hub genes. Zhai et al. collected data for 10,428 genes and obtained three modules associated with N staging, including 11 recurrence‐related genes.^19^ Using the Gene Expression Omnibus (GEO) database, Liu et al. screened staging‐related hub genes by WGCNA and further showed that these hub genes were correlated with DNA methylation and tumor‐infiltrating immune cells.^20^ Tang et al. used WGCNA to identify five hub genes related to the progression of PTC within GSE27155.^21^ Similarly, Ao et al. identified five candidate genes involved in the pathogenesis of PTC using the GSE33630 dataset.^22^

The screening of tumors for hub lncRNAs, circRNAs, and mRNAs by WGCNA can be used to predict potential regulatory networks based on interactions between non‐coding RNAs and mRNAs.^23^, ^24^, ^25^ In this study, the whole transcriptome of PTC was comprehensively analyzed for the first time by WGCNA, revealing potential interactions among circRNAs, lncRNAs, miRNAs, and mRNAs involved in disease progression. We explored the competitive endogenous RNAs in the regulation of protein‐coding genes and biological processes, thus providing insights into communication between RNAs during the development and progression of PTC.

In this study, five key modules were detected based on the WGCNA of whole transcriptome sequencing data. Among these clinical trait‐related modules, the gray module was related to metastasis in the lateral cervical lymph nodes. Moreover, we identified survival‐related hub genes, including *TMEM63C*, *COL5A1*, *LOXL1*, *ADAMTS2*, and *LYSMD3*. Using tissue samples and TCGA data, we further validated that *COL5A1* and *LOXL1* are overexpressed in PTC, which was consistent with the sequencing results. The Kaplan–Meier curve analyses supported the prognostic value of *COL5A1* and *LOXL1*.

COL5A1 belongs to the collagen family, is mainly expressed in the ECM, and is involved in cell adhesion and collagen remodeling. COL5A1 regulates the TGF‐β pathway, which is responsible for the poor prognosis.^26^ Liu et al. found that *COL5A1* is abnormally expressed in lung adenocarcinoma tissues, especially in advanced lung adenocarcinoma.^27^ In vitro experiments have shown that *COL5A1* is related to lung adenocarcinoma metastasis and may play a role in tumor regulation, proliferation, and progression.^27^ Our results showed that *COL5A1* is involved in PTC proliferation and migration, and its effects may be mediated by ECM receptor interactions. *COL5A1* is a potential prognostic marker for PTC and may function via ECM remodeling.

LOXL1 is a member of the lysyl oxidase family, which mainly includes copper‐dependent amine oxidases that catalyze the covalent cross‐linking of collagen and elastic fibers.^28^ *LOXL1*‐deficient mice are more likely to develop pelvic organ prolapse.^29^ In non‐small cell lung cancer (NSCLC), cancer‐associated fibroblast (CAF)‐secreted LOXL1 provides a microenvironment that facilitates tumor growth and invasion by affecting collagen fiber remodeling.^30^ This process is regulated by TGF‐β‐mediated integrin α11 in CAFs.^30^ Moreover, *LOXL1* expression is associated with chemoresistance in NSCLC and pancreatic ductal carcinoma.^31^, ^32^ In the present study, PTC cells with high *LOXL1* expression showed more aggressive behavior, consistent with the results obtained for other cancers.^30^ *LOXL1* can serve as a molecular marker for advanced PTC, and its specific role in the ECM requires further experiments.

Our WGCNA and experimental assays revealed that *COL5A1* and *LOXL1* are potential biomarkers for predicting aggressive biological behavior and prognosis in PTC. The functions of circRNAs and lncRNAs related to *COL5A1* and *LOXL1* in the turquoise module should be further explored.

This study had some limitations. The samples for RNA sequencing were all obtained from females and the sample size was small, which may have led to selection biases. Therefore, a larger sample size for tissue verification is required, and long‐term follow‐up data of our center are essential. Further studies of the expression of circRNAs, lncRNAs, miRNAs, and their interactions with mRNAs in the occurrence and development of PTC are needed.

## 5 CONCLUSION

In this study, we used whole transcriptome sequencing data for six pairs of PTC tissue samples to construct a gene co‐expression network based on circRNA/lncRNA–miRNA–mRNA interactions using WGCNA. Five survival‐related genes, *TMEM63C*, *COL5A1*, *LOXL1*, *ADAMTS2*, and *LYSMD3* were identified; the expression of *COL5A1* and *LOXL1* was upregulated in PTC tissues and may be related to OS and PFS of thyroid cancer patients. Furthermore, *COL5A1* and *LOXL1* promoted PTC cell proliferation and invasion in vitro. *COL5A1* and *LOXL1* are candidate survival biomarkers for PTC, and their interactions with circRNAs, lncRNAs, and miRNAs should be further explored.

## CONFLICT OF INTEREST

The authors declare that they have no conflicts of interest.

## AUTHOR CONTRIBUTION

Kexin Meng performed study concept and design and drafted the paper; Xiaotian Hu and Chenhong Qian performed experimental verification; Guowan Zheng and Ying Xin provided technical support; Ru He provided data analysis and interpretation; Haiwei Guo carried out the data collection; Minghua Ge participated in design and coordination; Jiajie Xu performed review and revision of the paper.

## CONSENT FOR PUBLICATION

Written informed consents were obtained from all the patients for publication of this case report.

## ETHICS STATEMENT

This study was approved by the Ethics Committee of Zhejiang Provincial People's Hospital and conducted in conformity to the Declaration of Helsinki.

## Supporting information


Table S1–S4
Figure S1–S3

## Data Availability

These sequence data have been submitted to the CNGB Sequence Archive (CNSA) of China National GeneBank DataBase (CNGBdb) with accession number CNP0002164 (https://db.cngb.org/cnsa/).
