# Supplementary material for: Identification of prognostic biomarkers for papillary thyroid carcinoma by a weighted gene co‐expression network analysis
Source: Cancer Med. 2022 Feb 12;11(9):2006–19. doi: 10.1002/cam4.4602 (PMC9089218; doi:10.1002/cam4.4602)
Supplement: Supplementary file 1 — Table S1–S4 Figure S1–S3 [file CAM4-11-2006-s001.docx]

**Supplementary figures and tables**

Table S1 sequences of forward and reverse primers for qRT-PCR

| **Gene** | **Primer, 5’-3’** | |
| --- | --- | --- |
|  | forward | reverse |
| *LYSMD3* | AGTTCAGTTCCTTGACCGAAAC | GCAAGAGAATCATTAGCTGGCA |
| *COL5A1* | GCCCGGATGTCGCTTACAG | AAATGCAGACGCAGGGTACAG |
| *LOXL1* | CTGTGCTGCGGAGGAGAAG | GTAGTGGCTGAACTCGTCCA |
| *TMEM63C* | GGAAAGCTGCGTGGGACTAT | TCCAAGGAAGTCTCCGAGGG |
| *ADAMTS2* | ATCACCAGGTCTTGGGAAGTT | AGTGAAGCGATAGTGGGTTCG |
| β-Actin | CATGTACGTTGCTATCCAGGC | CTCCTTAATGTCACGCACGAT |

Table S2 sense and antisense sequences of *COL5A1* siRNA and *LOXL1* siRNA

| **Gene** | **Primer, 5’-3’** | |
| --- | --- | --- |
|  | sense | antisense |
| siCOL5A1-1 | GAGAGGGUGAGACCUAUUATT | UAAUAGGUCUCACCCUCUCTT |
| siCOL5A1-2 | GGGAUUCCUUCAAGGUUUATT | UAAACCUUGAAGGAAUCCCTT |
| siCOL5A1-3 | UGAGACCUAUUACUACGAAUATT | UAUUCGUAGUAAUAGGUCUCATT |
| siRNA-NC | UUCUCCGAACGUGUCACGUTT | ACGUGACACGUUCGGAGAATT |
| siLOXL1-1 | CCUACAAUGCGGACAUCGATT | UCGAUGUCCGCAUUGUAGGTT |
| siLOXL1-2 | ACGUGGUGAGAUGCAACAUUCTT | GAAUGUUGCAUCUCACCACGUTT |
| siLOXL1-3 | CGCUACGUUUCUGCAACAAACTT | GUUUGUUGCAGAAACGUAGCGTT |

Table S3 Clinical information of patients

|  | Age | Sex | T stage | N stage | extrathyroid extension | multifocality | Vascular invasion | Type of surgery |
| --- | --- | --- | --- | --- | --- | --- | --- | --- |
| 1 | 30 | F | T1a | N1b | No | no | no | Total thyroidectomy |
| 2 | 38 | F | T3b | N1b | Yes | yes | no | Total thyroidectomy |
| 3 | 46 | F | T1b | N1b | Yes | no | no | Total thyroidectomy |
| 4 | 23 | F | T1b | N1a | No | no | no | lobectomy with isthmusectomy |
| 5 | 58 | F | T1b | N0 | No | no | no | lobectomy with isthmusectomy |
| 6 | 34 | F | T3b | N1a | yes | no | yes | Total thyroidectomy |

Table S4 Top 20 hub genes in turquoise, yellow, brown, blue and gray modules.

| module | Gene name | Gene type | Node Degree |
| --- | --- | --- | --- |
| turquoise | *CMTM3* | mRNA | 350 |
|  | *CDH11* | mRNA | 333 |
|  | *CD276* | mRNA | 332 |
|  | AC134312.5 | lncRNA | 330 |
|  | MSTRG.49905 | lncRNA | 329 |
|  | *PLAU* | mRNA | 326 |
|  | MSTRG.92322 | lncRNA | 324 |
|  | *KCNN4* | mRNA | 322 |
|  | *LOXL1* | mRNA | 321 |
|  | *VDR* | mRNA | 320 |
|  | *COL1A1* | mRNA | 319 |
|  | *COL5A1* | mRNA | 319 |
|  | *LAMP5* | mRNA | 317 |
|  | *AEBP1* | mRNA | 316 |
|  | *SRPX2* | mRNA | 315 |
|  | *CLEC11A* | mRNA | 314 |
|  | *COL3A1* | mRNA | 313 |
|  | *ADAMTS2* | mRNA | 313 |
|  | *KIAA1211* | mRNA | 313 |
|  | hsa-miR-382-5p | miRNA | 312 |
| yellow | LINC00710 | lncRNA | 23 |
|  | chr14:58457759\|58487166 | circRNA | 23 |
|  | chr7:72807066\|72807356 | circRNA | 22 |
|  | MSTRG.107803 | lncRNA | 21 |
|  | MSTRG.80424 | lncRNA | 20 |
|  | chr13:23353785\|23371165 | circRNA | 20 |
|  | chr3:47037666\|47062346 | circRNA | 19 |
|  | MSTRG.73441 | lncRNA | 18 |
|  | chr7:105040839\|105081797 | circRNA | 17 |
|  | MSTRG.103567 | lncRNA | 17 |
|  | chr5:80449591\|80474830 | circRNA | 16 |
|  | MSTRG.72798 | lncRNA | 16 |
|  | LINC02026 | lncRNA | 15 |
|  | MSTRG.81901 | lncRNA | 15 |
|  | MSTRG.150892 | lncRNA | 14 |
|  | *GPR17* | mRNA | 13 |
|  | *TMC3* | mRNA | 12 |
|  | MSTRG.92925 | lncRNA | 12 |
|  | RERE-AS1 | lncRNA | 12 |
|  | MSTRG.122056 | lncRNA | 12 |
| brown | MSTRG.33366 | lncRNA | 118 |
|  | MSTRG.86715 | lncRNA | 118 |
|  | MSTRG.104898 | lncRNA | 117 |
|  | MSTRG.14970 | lncRNA | 116 |
|  | MSTRG.68459 | lncRNA | 115 |
|  | MSTRG.115929 | lncRNA | 114 |
|  | MSTRG.159191 | lncRNA | 113 |
|  | MSTRG.76405 | lncRNA | 113 |
|  | MSTRG.119041 | lncRNA | 111 |
|  | AC022960.1 | lncRNA | 110 |
|  | MSTRG.114260 | lncRNA | 110 |
|  | MSTRG.121826 | lncRNA | 109 |
|  | MSTRG.121606 | lncRNA | 109 |
|  | MSTRG.42352 | lncRNA | 108 |
|  | MSTRG.30703 | lncRNA | 108 |
|  | MSTRG.153667 | lncRNA | 107 |
|  | MSTRG.23327 | lncRNA | 107 |
|  | MSTRG.130003 | lncRNA | 106 |
|  | MSTRG.139469 | lncRNA | 106 |
|  | MSTRG.138988 | lncRNA | 105 |
| blue | MSTRG.46964 | lncRNA | 153 |
|  | MSTRG.157563 | lncRNA | 152 |
|  | MSTRG.35516 | lncRNA | 152 |
|  | AP000462.2 | lncRNA | 148 |
|  | *VPREB3* | mRNA | 147 |
|  | *CCDC126* | mRNA | 146 |
|  | *PLEKHF2* | mRNA | 145 |
|  | AC234772.2 | lncRNA | 143 |
|  | *GMFB* | mRNA | 141 |
|  | MSTRG.7845 | lncRNA | 140 |
|  | AC116535.1 | lncRNA | 139 |
|  | MSTRG.8121 | lncRNA | 138 |
|  | chr13:108243532\|108251316 | circRNA | 137 |
|  | *RNF138* | mRNA | 134 |
|  | MSTRG.100053 | lncRNA | 130 |
|  | *LYSMD3* | mRNA | 127 |
|  | MSTRG.143871 | lncRNA | 126 |
|  | *FANCB* | mRNA | 125 |
|  | MSTRG.99679 | lncRNA | 125 |
|  | MSTRG.117238 | lncRNA | 125 |
| Gray | *PSAT1* | mRNA | 13 |
|  | *ARG2* | mRNA | 13 |
|  | *SLC7A11* | mRNA | 13 |
|  | *PUF60* | mRNA | 13 |
|  | *CADM3* | mRNA | 13 |
|  | *ERICH2* | mRNA | 13 |
|  | *TRIB3* | mRNA | 13 |
|  | *TSLP* | mRNA | 13 |
|  | *PHGDH* | mRNA | 13 |
|  | *HIST3H2A* | mRNA | 13 |
|  | *PDE1C* | mRNA | 13 |
|  | *ATF5* | mRNA | 13 |
|  | *TMEM63C* | mRNA | 12 |
|  | chr6:42822796\|42852197 | circRNA | 12 |
|  | chr15:49011731\|49019552 | circRNA | 3 |
|  | hsa-miR-190a-5p | miRNA | 3 |
|  | hsa-miR-335-5p | miRNA | 3 |
|  | *SCG2* | mRNA | 2 |
|  | AC018553.1 | lncRNA | 2 |
|  | *SIM2* | mRNA | 2 |

Figure S1 Expression profiles of circRNA, lncRNA, miRNA and mRNA in PTC. **A-D** The volcano plot visualized the expression of circRNA(**A**) , lncRNA(**B**), miRNA(**C**) and mRNA (**D**) between PTC tissues and adjacent non-neoplastic tissues, respectively.

Figure S2 Clustered heat maps displayed differentially expressed circRNA(**A**) , lncRNA(**B**), miRNA(**C**) and mRNA(**D**).

Figure S3 **A–E** Validation of expression patterns for five genes (*TMEM63C, COL5A1, LOXL1, ADAMTS2,* and *LYSMD3*) in papillary thyroid carcinoma samples by unpaired samples (505 cancer and 59 normal samples) from The Cancer Genome Atlas (TCGA) database. TC, thyroid cancer. NT, normal tissue.
